# Imaging the fate of CAR-T cells *in vivo*

**DOI:** 10.3389/fimmu.2026.1922326

**Published:** 2026-08-20

**Authors:** Hongyi Wang, Yan Yan, Yueping Li, Sihan Chen, Rui Ling, Jinfeng Zhu, Xiaju Cheng

**Affiliations:** 1 State Key Laboratory of Radiation Medicine and Protection, School of Radiation Medicine and Protection, Collaborative Innovation Center of Radiological Medicine of Jiangsu Higher Education Institutions, Soochow University, Suzhou, China; 2 Department of Radiology and Nuclear Medicine, Xuanwu Hospital, Capital Medical University, Beijing, China; 3 The Third Affiliated Hospital of Soochow University, Changzhou, China; 4 Biomedical Basic Research Center (BBRC) of Jiangsu Province, Suzhou, China

**Keywords:** CAR-T cells, cell interaction, distribution, molecular imaging, real-time activity

## Abstract

Despite the remarkable therapeutic efficacy of chimeric antigen receptor T (CAR-T) cell therapy in hematologic malignancies, its clinical translation in solid tumors remains impeded by challenges including insufficient intratumoral infiltration, tumor heterogeneity, and the immunosuppressive tumor microenvironment. Molecular imaging, which enables the visualization and quantitative analysis of the *in vivo* behavior of CAR-T cell*s*, serves as a critical prerequisite for advancing the clinical application of CAR-T cell therapy in solid tumors. This review summarizes recent advances in molecular imaging techniques for monitoring the *in vivo* fate of CAR-T cells, covering their dynamic distribution, tumor recognition, and real-time functional activity. Additionally, the potential clinical value and current limitations of these approaches are discussed. It is proposed that this interdisciplinary integration may improve the precision of therapeutic response assessment and facilitate the optimization of personalized treatment strategies in the context of CAR-T cell therapy.

## 1 Introduction

Tumor immunotherapy has witnessed significant advancement in recent years, with Chimeric Antigen Receptor T (CAR-T) cell therapy emerging as a promising therapeutic modality that has exhibited notable clinical efficacy in hematological malignancies (1–3). To date, several CAR-T cell products have been approved by the U.S. Food and Drug Administration (FDA) for the treatment of hematologic malignancies. However, the clinical translation of CAR-T cell therapy to solid tumors is still constrained by multiple critical obstacles, including tumor antigen heterogeneity, limited tumor infiltration, and the immunosuppressive tumor microenvironment (TME), which collectively result in a substantially reduced therapeutic efficacy relative to that observed in hematologic cancers (4). Further investigations have revealed that, following intravenous infusion, CAR-T cells preferentially home to secondary lymphoid organs, where they undergo expansion and activation prior to migrating to tumor sites. Subsequently, they traverse vascular barriers, infiltrate tumor tissues, and ultimately recognize and eliminate tumor cells with high specificity. These observations indicate that the *in vivo* migration, infiltration, tumor antigen-specific recognition, and tumor-killing activity of CAR-T cells are closely correlated with their anti-tumor efficacy (5). However, in clinical practice, physicians are unable to obtain such information in a timely manner, which increases the uncertainty regarding patient responsiveness to CAR-T cell therapy.

Molecular imaging techniques enable noninvasive, dynamic, and quantitative *in vivo* monitoring of CAR-T cells, thereby providing critical tools for investigating their biological behaviors (6, 7). While prior reviews have summarized certain tracer-based imaging strategies for CAR-T cells, a systematic overview focusing on the dynamic behaviors of CAR-T cells within the TME in the context of solid tumor therapy and their association with therapeutic efficacy remains absent. Notably, the *in vivo* fate of CAR-T cells, encompassing dynamic distribution, tumor recognition, and real-time activity, has increasingly been recognized as a key determinant of treatment response. This review aims to summarize recent advances in molecular imaging approaches for characterizing these *in vivo* fates of CAR-T cells in solid tumor treatment, with particular emphasis on their potential roles in elucidating therapeutic barriers, predicting treatment efficacy, and guiding personalized therapeutic strategies. Additionally, we discuss the potential clinical value and current limitations of these imaging probes, with the objective of facilitating the clinical translation of these cross-disciplinary immunoimaging agents for precision cell therapies.

## 2 CAR-T cell therapy

**Figure 1:**
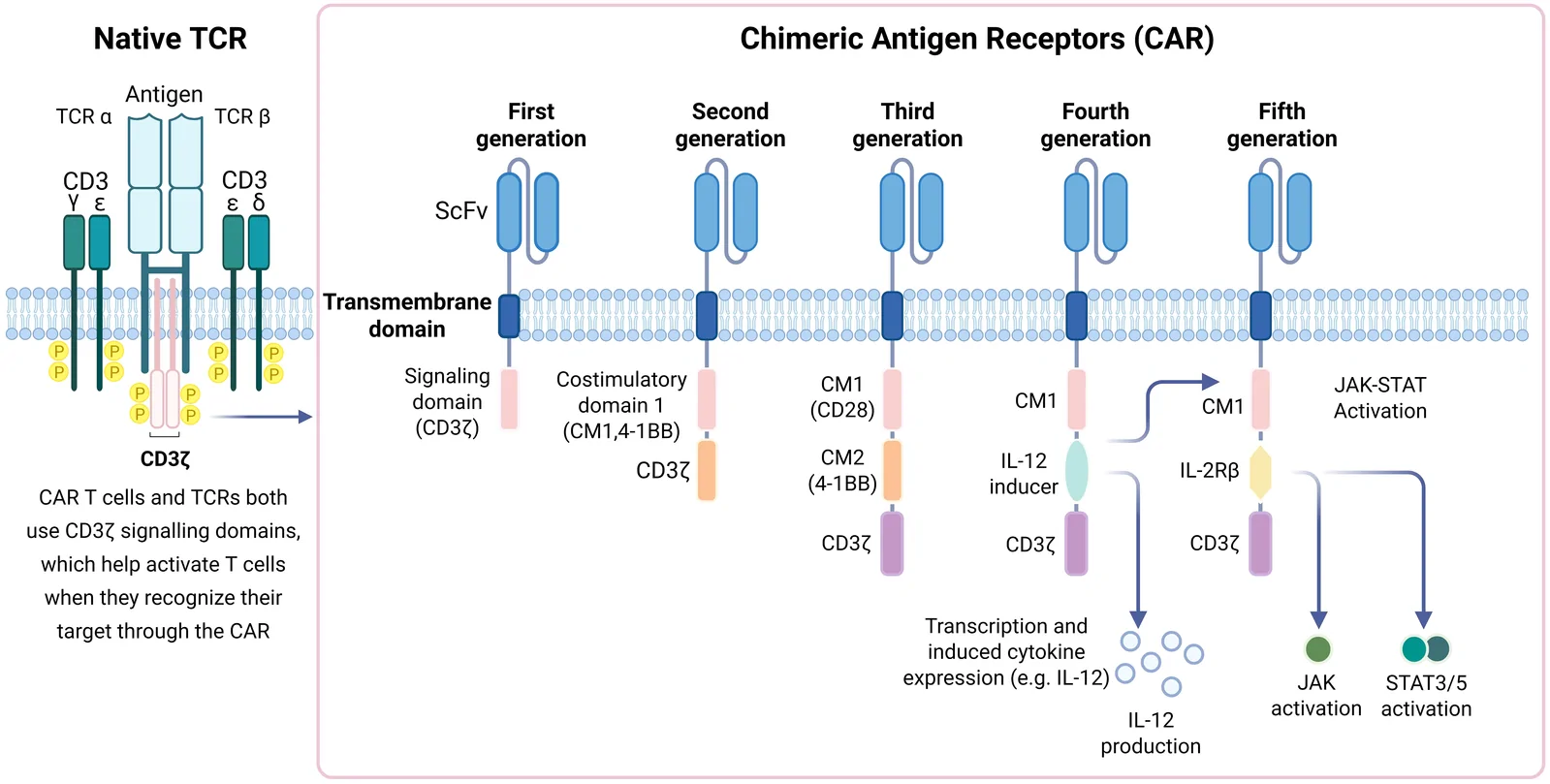
Generations of CAR-T cells. This figure illustrates the evolutionary trajectory of CAR-T cell generations, with comparative analysis against the structural framework of the T-cell receptor (TCR). CD3, Cluster of Differentiation 3; ScFv, Single-chain Variable Fragment; CM1, Costimulatory Molecule 1; 4-1BB, 4-1BB ligand receptor/CD137; IL-12, Interleukin-12; JAK, Janus Kinase; STAT3/5, Signal Transducer and Activator of Transcription 3/5. Figure created using BioRender (biorender.com).

CARs are synthetically engineered receptors comprising three core structural components: an extracellular antigen-recognition domain, a transmembrane domain, and an intracellular signaling domain (8). The extracellular segment typically includes a single-chain variable fragment (scFv) linked to a hinge/spacer sequence, which mediates target antigen binding and confers structural flexibility to facilitate antigen engagement (9). The transmembrane domain anchors the receptor to the cell membrane, while the intracellular domain transduces activation signals and initiates downstream effector functions (10). Thus, the structural design of CARs directly modulates antigen recognition specificity, signaling intensity, cellular persistence, and anti-tumor activity. Based on variations in their intracellular signaling architecture, CAR-T cells are commonly categorized into five generations (11, 12). This classification system reflects the progressive evolution of CAR designs, from simple antigen-triggered activation modules to complex constructs optimized for enhanced T-cell persistence, effector function, and resistance to immunosuppressive tumor microenvironments (**Figure 1**). First-generation CARs contain only the CD3ζ signaling domain; although capable of mediating antigen recognition and T-cell activation, their lack of costimulatory signaling results in limited proliferation, short-term persistence, and suboptimal anti-tumor efficacy (13). To address these limitations, second-generation CARs integrate a single costimulatory domain (most frequently CD28 or 4-1BB) alongside the CD3ζ domain, thereby improving T-cell expansion, survival, and cytotoxic activity (14, 15). Due to the balance between structural simplicity and functional potency, second-generation CARs remain the dominant platform in contemporary clinical applications. Building on this framework, third-generation CARs integrate two costimulatory domains to further potentiate intracellular activation signals, with the potential to enhance therapeutic efficacy (16). Subsequent advanced designs transition from mere amplification of CAR signaling to active modulation of the TME. Fourth-generation CARs, also referred to as TRUCKs, are genetically engineered to secrete immune-modulating molecules such as IL-12 or IL-15, with the objective of improving CAR-T cell functionality under immunosuppressive conditions (17). The most recent advancement, fifth-generation CARs, further extend the design principles of second-generation CARs by incorporating signaling modules derived from cytokine receptors, particularly components associated with the IL-2R/JAK–STAT pathway, thereby facilitating antigen-dependent proliferation, persistence, and maintenance of functional activity (18).

The trafficking, infiltration, and specific recognition of tumor cells by CAR-T cells constitute the mechanistic basis for their notable therapeutic efficacy, which has been most extensively validated in hematologic malignancies including acute lymphoblastic leukemia (ALL), diffuse large B-cell lymphoma (DLBCL), and multiple myeloma (MM) (19). Currently, clinically approved CAR-T cell products in both the United States and China are primarily focused on two core targets: CD19 and B-cell maturation antigen (BCMA) (20). Tisagenlecleucel (Kymriah), the first CAR-T cell therapy approved by the FDA, targets CD19 and integrates 4-1BB costimulatory signaling. In pediatric and young adult patients with relapsed or refractory B-ALL, a single infusion yielded high remission rates, with the majority of responders achieving deep molecular remission and exhibiting evidence of *in vivo* CAR-T cell persistence (21, 22). For aggressive B-cell lymphomas, CD19-directed CAR-T products such as axicabtagene ciloleucel and lisocabtagene maraleucel have also induced durable clinical responses in heavily pretreated patient cohorts (23, 24). More recently, BCMA-targeted CAR-T cell therapies have expanded the clinical application of this therapeutic modality to MM (25). Beyond the aforementioned approved indications, representative investigational CAR-T cell therapy strategies indicate that additional targets may exhibit high relevance in specific disease contexts. These targets include CD7 and CLL1/CD33-related targets for T-cell acute lymphoblastic leukemia/lymphoblastic lymphoma (T-ALL/LBL) and acute myeloid leukemia (AML), CLDN18.2 for gastric and pancreatic cancers, GPC3 for hepatocellular carcinoma (HCC), GD2 for neuroblastoma, and HER2, EGFRvIII, or B7-H3 for selected solid tumors such as glioblastoma and pediatric or young adult non-central nervous system (non-CNS) tumors (**Table 1**). Notably, malignant cells in hematological malignancies are generally more accessible within the circulation and lymphoid organs, target antigens such as CD19 and BCMA are relatively well-characterized, and the TME is typically less structurally restrictive compared to that of solid tumors (26).

**Table 1:** Approved and representative investigational CAR-T cell products.

Product/candidate	Target antigen(s)	Principal indication(s)	Country/regulatory authority	Initial approval year/clinical phase
Kymriah	CD19	B-cell precursor ALL (≤25 y); adult r/r LBCL or FL	United States - FDA	2017
Yescarta	CD19	Adult LBCL; r/r FL	United States – FDA	2017
Tecartus	CD19	Adult r/r MCL; r/r B-cell precursor ALL	United States – FDA	2020
Breyanzi	CD19	Adult r/r LBCL, CLL/SLL, FL, MCL, or MZL	United States – FDA	2021
Abecma	BCMA	Adult r/r MM	United States – FDA	2021
Carvykti	BCMA	Adult r/r MM	United States – FDA	2022
Aucatzyl	CD19	Adult r/r B-cell precursor ALL	United States - FDA	2024
Yikaida	CD19	Adult r/r LBCL	China, NMPA;	2021
Carteyva	CD19	Adult r/r LBCL, FL, or MCL	China, NMPA;	2021
Fucaso	BCMA	Adult r/r MM	China, NMPA;	2023
Carvykti	BCMA	Adult r/r MM	China, NMPA;	2025
Kailimei/satri-cel	CLDN18.2	CLDN18.2^+^/HER2^-^ advanced gastric or GEJ adenocarcinoma	China, NMPA;	2026
AZD0120	BCMA/CD19	Adult r/r MM	Multinational	Phase 1b/2 (NCT05850234); Phase 3 (NCT07391657)
CD19/CD22 dual CAR-T programs	CD19/CD22	Pediatric/young adult r/r B-cell malignancies	United States	Phase 1 (NCT03448393); Phase 1/2 (NCT05442515)
CD7 CAR-T	CD7	r/r T-ALL/T-LBL;	Multinational	Phase 1 (NCT04984356); Phase2 (NCT06514794)
CLL1-targeted CAR-T	CLL1	r/r AML or MDS	United States	Phase 1/2 (NCT06680752)
GPC3 CAR-T	GPC3	HCC/other GPC3-positive solid tumors	United States and China	Phase 1 (NCT05003895; NCT03980288)
B7-H3 CAR-T	B7-H3	Pediatric/young adult r/r B7-H3^+^ non-CNS solid tumors	United States	Phase 1 (NCT04483778)
GD2 CAR-T/GD2-CART01	GD2	High-risk or r/r neuroblastoma and other GD2^+^ solid tumors	United States and Italy	Phase 1 (NCT02107963); Phase 1/2 (NCT03373097)
HER2 CAR-T	HER2	Recurrent/progressive HER2^+^ glioblastoma	United States	Phase 1 (NCT01109095)
EGFRvIII CAR-T	EGFRvIII	EGFRvIII^+^ malignant glioma	United States	Phase 1/2 (NCT01454596)

*ALL, acute lymphoblastic leukemia; LBCL, large B-cell lymphoma; FL, follicular lymphoma; CLL, chronic lymphocytic leukemia; SLL, small lymphocytic lymphoma; MCL, mantle cell lymphoma; MM, multiple myeloma; LBL, Lymphoblastic Lymphoma; AML, acute myeloid leukemia; MDS, myelodysplastic syndrome; HCC, hepatocellular carcinoma; CNS, central nervous system; r/r, relapsed/refractory.

By contrast, the translation of CAR-T cell therapy to solid tumors remains considerably more challenging (27). Post-infusion, CAR-T cells are required to undergo systemic distribution, activation, expansion, trafficking to tumor sites, transvascular extravasation, tumor parenchyma infiltration, and ultimate tumor cell recognition and (28) elimination. However, each of these sequential steps may be compromised by insufficient trafficking, suboptimal intratumoral infiltration, antigen heterogeneity, and the profoundly immunosuppressive TME (29). In response to these barriers, certain clinical studies have endeavored to enhance tumor accessibility by employing locoregional rather than exclusive systemic delivery strategies. A representative example is the IL13Rα2-targeted CAR-T trial for recurrent or refractory malignant glioma, which evaluated intratumoral, intracavitary, intraventricular, and dual-administration routes (ClinicalTrials.gov identifier: NCT02208362). Thus, despite substantial improvements in T-cell potency achieved via CAR engineering, the therapeutic efficacy of CAR-T cells in solid tumors remains contingent on their ability to successfully execute the complete *in vivo* trafficking–recognition–effector cascade. Collectively, the development of CAR-T cell therapy reflects an evolutionary trajectory from optimizing receptor design to enhancing *in vivo* persistence and resistance to hostile microenvironments. While clinical success in hematologic malignancies has validated the therapeutic potential of this approach, the limited efficacy observed in solid tumors underscores the necessity of deeper mechanistic insights into the *in vivo* trafficking, infiltration, target recognition, and functional dynamics of CAR-T cells.

## 3 Challenges of CAR-T cell therapy in solid tumors

**Figure 2:**
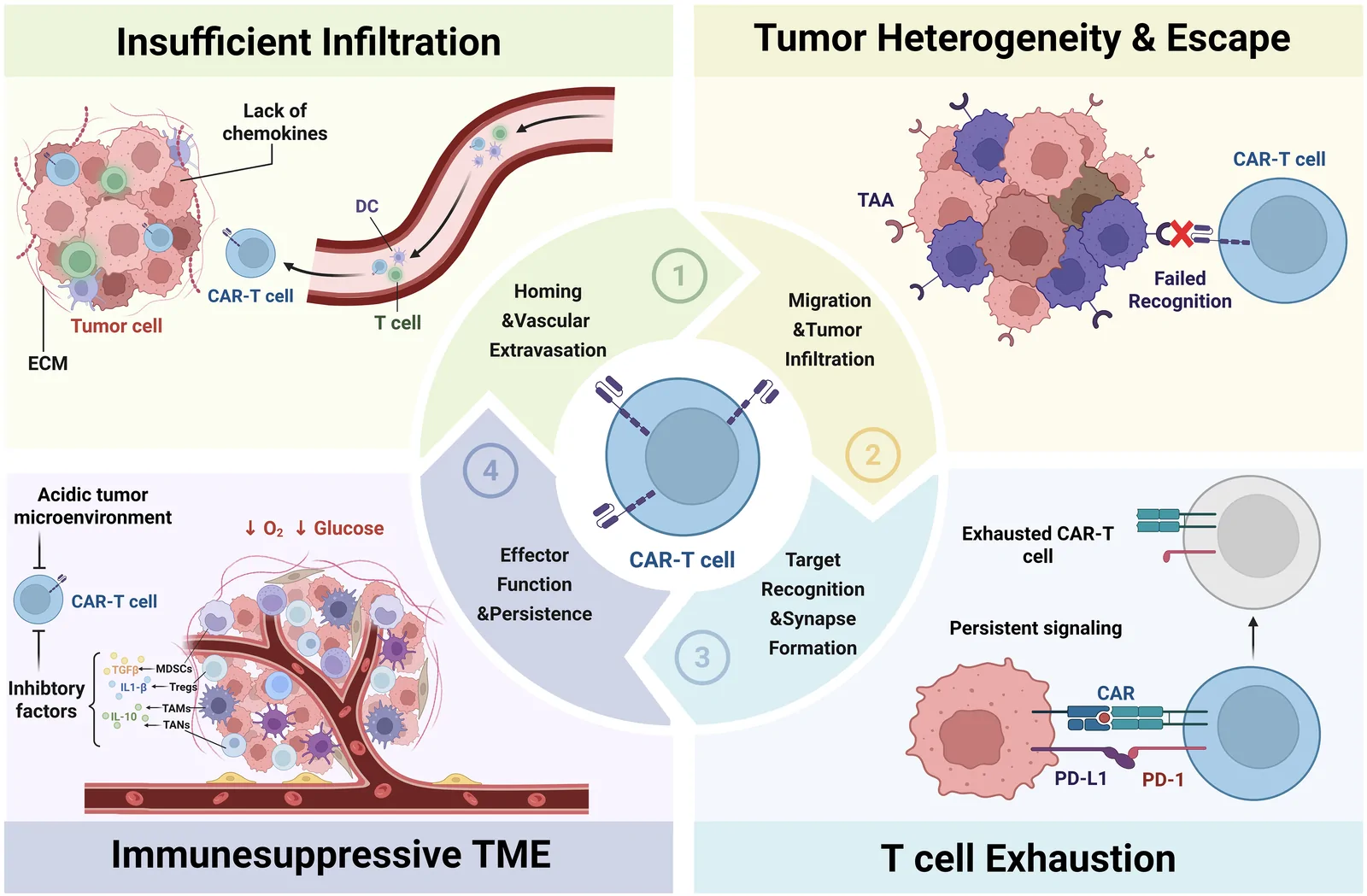
Challenges of CAR-T cell therapy in solid tumors. CAR-T cell therapy for solid tumors is confronted with four primary barriers: (1) inefficient tumor homing and extravasation; (2) restricted infiltration across dense stromal/ECM barriers; (3) antigen heterogeneity that impairs tumor recognition; and (4) immunosuppressive TME that induce T-cell dysfunction and exhaustion. DC, dendritic cell; TAA, tumor-associated antigen; MDSCs, myeloid-derived suppressor cells; TAMs, tumor-associated macrophages; TANs, tumor-associated neutrophils; PD-L1, programmed death-ligand 1; PD-1, programmed cell death-1. Figure created using BioRender (biorender.com).

The therapeutic efficacy of CAR-T cells in solid tumors relies on a sequential cascade of events involving systemic trafficking, tumor localization, and sustained effector function (30). Following systemic administration, CAR-T cells circulate in the bloodstream and must achieve effective homing to tumor sites, a process regulated by chemokine gradients and adhesion molecule interactions with vascular endothelial cells (31). Subsequently, they undergo firm adhesion and extravasation across the tumor vasculature, followed by infiltration into the tumor parenchyma, where they migrate within a complex microenvironment composed of stromal components and extracellular matrix (ECM) (32, 33). Upon encountering tumor cells, CAR-T cells recognize target antigens via CAR-mediated binding, form immunological synapses, and exert cytotoxic effects through perforin/granzyme release and cytokine secretion. Sustained antitumor efficacy further necessitates the persistence, proliferation, and resistance to functional exhaustion of CAR-T cells within the TME (34). Notably, this sequential cascade is highly vulnerable to disruption at multiple nodes in solid tumors. Efficient tumor homing relies on the interaction between tumor-derived chemokines (e.g., CXCL9/10 and CCL5) and their cognate receptors on CAR-T cells, whereas aberrant vasculature and endothelial barriers may impede extravasation. Within the tumor parenchyma, dense ECM structures and stromal components restrict T-cell migration and spatial distribution. Even following successful infiltration, CAR-T cells must contend with antigen heterogeneity and immunosuppressive signals that compromise their functional activity (30, 35). Collectively, these barriers indicate that the limited efficacy of CAR-T cell therapy in solid tumors arises not from a single defect, but from cumulative impairments across multiple spatial and functional stages of the trafficking–recognition–effector axis (36) (**Figure 2**).

### 3.1 Inefficient infiltration of CAR-T cells in tumors

In solid tumors, CAR-T cells frequently encounter challenges in transendothelial migration across the vascular endothelium to infiltrate tumor tissue (32). Following systemic administration, CAR-T cells face significant obstacles to penetrating the solid tumor parenchyma (37). Aberrant tumor vasculature, elevated interstitial fluid pressure, and a dense ECM restrict T-cell extravasation and intratumoral trafficking, thereby reducing the number of effector cells that reach tumor nests (38). Additionally, many solid tumors exhibit insufficient expression of T-cell-recruiting chemokines (e.g., CCL4/CCL5 and CXCL9/CXCL10), which impairs lymphocyte homing and further limits CAR-T cell accumulation within the lesion (39). The prevalence of activated cancer-associated fibroblasts and a desmoplastic, fibrotic stroma generates both a physical meshwork and biochemical signals that hinder CAR-T cell migration and functional activity (27).

### 3.2 Insufficient tumor recognition induced by tumor heterogeneity

Tumor antigen heterogeneity constitutes a critical barrier to the efficacy of CAR-T cell therapy in solid tumors. In contrast to hematologic malignancies, which typically express lineage-specific antigens with relatively high homogeneity, solid tumors exhibit pronounced intra- and intertumoral heterogeneity in antigen expression. This variability not only reduces the proportion of tumor cells susceptible to targeted elimination but also fosters niches for the persistence and proliferation of antigen-negative subclones (36). Moreover, antigen escape, defined as the downregulation or complete loss of the targeted antigen, further exacerbates this limitation. Under the selective pressure imposed by CAR-T cell therapy, tumor cells may undergo adaptive modulation of antigen expression, thereby evading immune recognition and cytotoxicity (33). This phenomenon has been extensively documented in hematological malignancies, where relapse frequently occurs following CD19-targeted CAR-T cell therapy due to antigen loss or splice variants. In solid tumors, where antigen expression is inherently heterogeneous, antigen escape presents an even more formidable challenge, severely compromising the durability of therapeutic responses.

### 3.3 Immunosuppressive tumor microenvironment

The immunosuppressive and structurally complex TME constitutes a critical distinguishing feature of solid tumors relative to hematological malignancies (40, 41). Myeloid and regulatory cell populations, including tumor-associated macrophages (TAMs), myeloid-derived suppressor cells (MDSCs), and regulatory T cells (Tregs), secrete inhibitory mediators (e.g., TGF-β, IL-10, ARG1, and IDO) that attenuate T-cell activation and proliferation. The dense desmoplastic stroma, driven by cancer-associated fibroblasts and cross-linked ECM, forms a physical barrier that restricts the access of CAR-T cells to tumor nests. Abnormal vasculature, elevated interstitial pressure, and local hypoxia further impair the extravasation and intratumoral trafficking of effector cells (42). Spatial profiling studies have revealed immunosuppressive gradients within tumor lesions, with central tumor regions typically exhibiting the highest hostility toward cytotoxic lymphocytes; this regional suppression limits the accumulation and functional activity of CAR-T cells (43).

### 3.4 T cell exhaustion

Exhaustion of CAR-T cells constitutes a critical bottleneck in the advancement of effective CAR-T cell therapies, which is a progressive and hierarchical state defined by the sustained loss of effector functions recognized as a central mechanism underpinning therapeutic failure and disease recurrence (44). Mechanistically, T cell exhaustion encompasses not only surface inhibitory signaling cascades but also profound intracellular reprogramming events, including: (1) transcriptional rewiring driven by key regulators such as thymocyte selection-associated high mobility group box (TOX) and nuclear factor of activated T cells (NFAT); (2) metabolic dysregulation characterized by compromised mitochondrial fitness and reduced glycolytic capacity; and (3) progressive functional decline marked by diminished cytokine secretion and cytotoxic activity. Both tonic CAR signaling and chronic antigen stimulation further accelerate the trajectory of exhaustion. Moreover, solid tumors impose additional metabolic and environmental constraints on CAR-T cells, such as hypoxic, acidic, and nutrient-deprived conditions within the TME. These factors disrupt cellular metabolic homeostasis, elevate oxidative stress, and further exacerbate exhaustion, ultimately compromising CAR-T cell persistence and anti-tumor efficacy (45).

## 4 *In vivo* monitoring of CAR-T cells via molecular imaging

Given the multistep biological processes underpinning effective CAR-T cell therapy for solid tumors, molecular imaging should not be confined to verifying the presence of transferred CAR-T cells at tumor sites. Instead, contemporary imaging strategies can be systematically categorized into three complementary dimensions of T cell behavior: spatial distribution, tumor recognition, and functional activity. For instance, cell-tracking approaches leveraging various imaging modalities enable visualization of CAR-T cell trafficking, biodistribution, tumor homing, and intratumoral infiltration. Besides, emerging imaging strategies targeting T cell-tumor cell interactions offer insights into whether accumulated T cells can physically engage tumor cells and initiate functionally relevant immune events. Finally, function-oriented imaging approaches further evaluate whether T cells exhibit metabolic activation, phenotypic engagement, or effector function execution. These imaging strategies advance the field beyond the simplistic inquiry of “where the cells are” to a more comprehensive assessment addressing “whether they reach the tumor, recognize their targets, and maintain functional activity.” Below, we summarize current molecular imaging technologies in the context of these core biological questions regarding *in vivo* CAR-T cell behavior.

### 4.1 Imaging for CAR-T cell tracking

**Figure 3:**
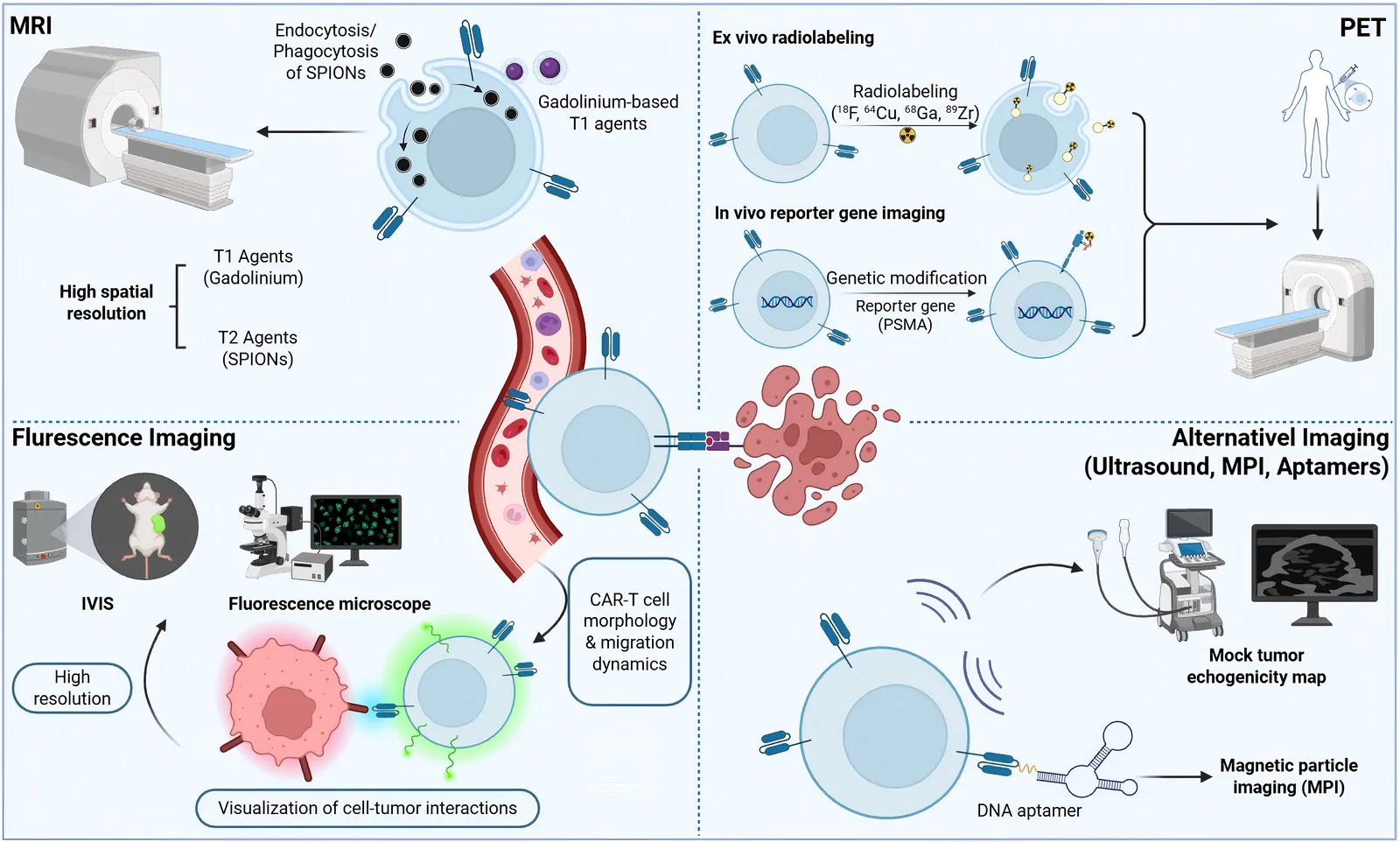
Molecular imaging for monitoring CAR-T cell tracking. Current molecular imaging modalities, including magnetic resonance imaging (MRI), positron emission tomography (PET), fluorescence imaging, magnetic particle imaging (MPI), and ultrasound imaging, enable noninvasive visualization of CAR-T cell trafficking, tumor infiltration, and functional dynamics *in vivo*. IVIS, *In Vivo* Imaging System. Figure created using BioRender (biorender.com).

Molecular imaging provides a robust noninvasive approach for visualizing and quantifying CAR-T cell trafficking and biodistribution in real time. By delivering spatial and temporal data, these techniques enable a more comprehensive assessment of treatment outcomes and facilitate the identification of barriers to therapeutic efficacy. These insights are essential for elucidating the mechanisms underlying treatment failure and guiding the optimization of CAR-T cell therapies, especially in solid tumors. In this section, we review the major molecular imaging modalities employed for *in vivo* CAR-T cell tracking and discuss their applications, advantages, and limitations in the context of CAR-T cell therapy (**Figure 3**).

#### 4.1.1 MRI

Owing to its capacity for visualizing deep tissues with high spatial resolution and without ionizing radiation, magnetic resonance imaging (MRI) holds significant value in the diagnosis of solid tumors (46). Currently, MRI contrast agents employed for CAR-T cell tracking primarily encompass gadolinium-based contrast agents (GBCAs), fluorine-19 MRI (^19^F MRI) probes, and superparamagnetic iron-oxide nanoparticles (SPIONs). Among these, GBCAs (e.g., gadolinium chelates and gadolinium-containing nanoparticles) and ^19^F MRI probes (e.g., perfluorocarbon nanoemulsions, PFCs) are internalized by CAR-T cells through endocytosis and pinocytosis, thereby enabling noninvasive MRI visualization of labeled CAR-T cells (47, 48). In contrast, SPIONs can be delivered into cells via multiple pathways, including phagocytosis, receptor-mediated endocytosis (e.g., transferrin targeting), translocation peptides (e.g., HIV TAT peptide), polycationic transfection reagents (e.g., poly-L-lysine, Lipofectamine, protamine sulfate), and electroporation (49).

For instance, Zhang et al. demonstrated that adoptive T cells could be efficiently labeled with NaGdF_4-_TAT nanoprobes, allowing sensitive detection of labeled T-cell clusters in orthotopic glioma models *in vivo* using T1-weighted imaging (T1WI) (50). This strategy underscores the potential of MRI-based T-cell tracking for future personalized immunotherapy in glioma patients. Building on prior research, Xie et al. labeled CAR-T cells with ultra-small superparamagnetic iron oxide particles (USPIOs), enabling quantitative MRI tracking of CAR-T cell infiltration and persistence in glioblastoma models (51). The results indicated that increasing hypointense signals were detectable in glioblastoma models up to 14 days following the injection of USPIO-labeled CAR-T cells. Similarly, Kiru et al. labeled CAR-T cells with iron oxide nanoparticles (ferumoxytol) using a microfluidics-based mechanoporation approach, facilitating noninvasive multimodal imaging *via* MRI, photoacoustic imaging (PAT), and magnetic particle imaging (MPI) in osteosarcoma models (52). The results demonstrated successful *in vivo* visualization of CAR-T cell homing to osteosarcoma. However, ferumoxytol has been associated with rare but severe anaphylactic reactions. To address this concern, Wu et al. developed MegaPro nanoparticles (MegaPro-NPs) with improved safety profiles for *in vivo* tracking of CAR-T cells in preclinical glioblastoma models (53). Further extending the application of MRI in CAR-T cell tracking, Dubois et al. employed ^19^F MRI to visualize CAR-T cells in a murine leukemia model (54). By labeling the cells with ^19^F PFC, they demonstrated that ^19^F MRI could be used to visualize PFC-labeled CAR-T cells for up to 7 days post-intratumoral injection without significantly affect *in vivo* CAR-T cell cytotoxicity. In summary, MRI can visualize CAR-T cell localization and tumor infiltration with high spatial resolution. However, low sensitivity, signal dilution during cell proliferation, and limited viability assessment restrict its application in long-term monitoring (55).

#### 4.1.2 PET

PET, characterized by its high sensitivity and quantitative capacity, enables the detection of small populations of labeled cells and longitudinal monitoring of CAR-T cell trafficking, expansion, and persistence *in vivo* (56). Current PET-based CAR-T cell tracking strategies can be broadly categorized into ex vivo radiolabeling and *in vivo* reporter gene imaging (57). In ex vivo radiolabeling, CAR-T cells are labeled with positron-emitting radionuclides, most commonly ^18^F, ^64^Cu, ^68^Ga, and ^89^Zr, prior to infusion. These radionuclides are typically introduced into cells via lipophilic carrier complexes that enhance intracellular retention. For example, Weist et al. validated the efficacy of ^89^Zr-oxine labeling of CAR-T cells without impairing their viability or function, facilitating longitudinal tracking in murine glioblastoma and prostate cancer models via PET (58). Additionally, Pan, D. et al. pioneered the labeling of anti-CD19 CAR-T cells with [^68^Ga]Ga-DOTA-biotin in Raji hematologic tumor models, enabling real-time monitoring of CAR-T cell proliferation *in vivo* (59). The activity and cytotoxicity of the modified CAR-T cells remained uncompromised. They further developed an ex vivo labeling strategy involving direct labeling of CAR-T cells with ^68^Ga-oxine, which enabled noninvasive PET imaging for *in vivo* pharmacokinetic and pharmacodynamic analyses. Notably, ^68^Ga-labeled CAR-T cells exhibit an approximately 24-fold lower absorbed dose compared to ^89^Zr-labeled cells, indicating enhanced safety and supporting their potential for clinical translation (60, 61).

In contrast, *in vivo* labeling enables longitudinal PET imaging of infused CAR-T cells by using radiotracers that target a reporter gene co-expressed with the CAR construct or a genetically introduced cell-surface marker. Unlike direct radiolabeling, reporter gene strategies allow repeated imaging without signal dilution caused by radionuclide decay or cell proliferation, thereby providing quantitative assessment of CAR-T cell trafficking, persistence, and tumor engagement over time. For instance, Minn I. et al. demonstrated that CD19-tPSMA^(N9del)^ CAR-T cells could be tracked using [^18^F]DCFPyL PET in a Nalm6 model of acute lymphoblastic leukemia (62). This approach allows noninvasive, serial imaging to quantitatively assess CAR-T cell distribution, persistence, and tumor engagement. Similarly, Song, X. et al. engineered transferrin receptor-ΔPSMA CAR (CAR-ΔPSMA) T cells, which could be successfully tracked *in vivo* using [^68^Ga]Ga-PSMA-617 via PET/CT in a breast cancer model (63). Importantly, PSMA reporter expression preserved CAR-T cell function while enabling sensitive longitudinal PET imaging of tumor accumulation and persistence. Other reporter genes, such as herpes simplex virus thymidine kinase (HSV-TK), have also demonstrated promise for long-term cell tracking. These findings highlight the need for noninvasive longitudinal monitoring of CAR T-cell dynamics in clinical settings. Moreover, these studies illustrate the potential of PET to elucidate CAR-T cell behavior *in vivo*, offering important guidance for future therapy design. However, current evidence also indicates that CAR-T cells show limited infiltration into solid tumors, resulting in suboptimal therapeutic efficacy.

#### 4.1.3 Fluorescence imaging

While PET and MRI are increasingly recognized as the leading modalities for clinical translation of CAR-T cell imaging, fluorescence-based approaches continue to play a central role in mechaStic and preclinical investigations (64, 65). Their widespread use stems from the ability to sensitively visualize CAR-T cell trafficking with relatively simple experimental procedures and low operational costs. In particular, NIR fluorescence imaging has been extensively employed to monitor CAR-T cell migration, tumor homing, and local accumulation in small-animal models. For instance, Harmsen et al. developed a dual-modal PET/NIR fluorescent Nanotag for non-genomic labeling of human CAR-T cells and demonstrated long-term whole-body tracking in an ovarian peritoneal carcinomatosis model (65). More recently, He et al. labeled CLDN18.2-targeted CAR-T cells with both SPIONs and the NIR dye DiR, enabling dynamic observation of CAR-T cell biodistribution and tumor accumulation in a gastric cancer model through MPI/FMI dual-modality imaging (66). Furthermore, Yan et al. established an optically transparent immunocompromised zebrafish platform co-engrafted with fluorescently labeled human tumor cells and CAR-T cells, enabling real-time single-cell imaging of T-cell immunotherapy responses *in vivo* (67). These studies suggest that fluorescence imaging is valuable for monitoring CAR-T cell homing and tumor localization but is often used in combination with other imaging modalities.

To improve CAR-T cell tracking, fluorescence imaging is increasingly combined with reporter systems and other imaging modalities. For instance, Zhang et al. combined optical imaging with PSMA-targeted PET to support the development of an *in situ* CAR-T cell protocol, illustrating how optical signals can complement nuclear imaging for monitoring CAR-T generation, biodistribution, and tumor engagement *in vivo* (68). Together, fluorescence imaging provides a simple and sensitive approach for studying CAR-T cell behavior in preclinical models, although its limitations in tissue penetration and quantification often necessitate multimodal imaging strategies.

#### 4.1.4 Other imaging modalities

Recent methodological innovations epitomize the ongoing endeavor to address the limitations of traditional modalities while expanding the scope of molecular imaging technologies. Huang et al. engineered gas vesicle-labeled CAR-T cells (GVs@CAR-T) for ultrasound-based *in vivo* tracking (69). The labeled cells maintained visualization for up to 5 days without impairing anti-tumor function, enabling real-time monitoring of CAR-T cell tumor infiltration and serving as a valuable tool for therapeutic efficacy assessment. Similarly, magnetic particle imaging (MPI) has emerged as a sensitive and quantitative modality for cell tracking. Meng et al. labeled CLDN18.2-targeted CAR-T cells with superparamagnetic iron oxide nanoparticles (SPIONs) and demonstrated dynamic monitoring of their migration, tumor accumulation, and persistence in gastric cancer xenografts (66). The significantly higher MPI signal in tumors receiving SPION-labeled CAR-T cells compared to those receiving labeled normal T cells confirmed antigen-specific *in vivo* tumor targeting. Furthermore, the integration of MPI with fluorescence imaging facilitated multimodal assessment of CAR-T cell behavior and response to combination immunotherapy.

Collectively, these emerging imaging platforms enable near-real-time monitoring of CAR-T cell migration and tumor infiltration, capabilities challenging to achieve with MRI and PET. Moreover, they provide functional readouts beyond localization, including infiltration dynamics, homing behavior, and correlations with therapeutic response, thereby enhancing the *in vivo* evaluation of CAR-T cell therapy and supporting its application in solid tumors (**Table 2**).

**Table 2:** Molecular imaging strategies for monitoring CAR-T cell tracking.

Modality	Strategy/probe	Tracked cells	Experimental model	Refs
MRI	NaGdF_4_-TAT nanoparticles	Adoptive T cells	GBM in mice	(50)
	SPION-labeled CAR-T cells	EGFRvIII/IL13 Rα2 CAR-T cells	GBM in mice	(51)
	Ferumoxytol-labeled CAR-T cells	CAR-T cells	OS in mice	(52)
	MegaPro-NP-labeled CAR-T cells	CAR-T cells	GBM in mice	(53)
	^19^F PFC labeling	CAR-T cells	murine leukemia model	(54)
PET	^89^Zr-oxine	huLym-1-A-BB3z CAR-T cells	GBM, prostate cancer, and lymphoma models in mice	(58)
	[^68^Ga]Ga-DOTA-biotin	SA-CD19-CAR-T cells	Raji lymphoma xenograft in NSG mice	(59)
	Direct ^68^Ga labeling	CD19 CAR-T cells	K562 and Raji tumors in NSG mice	(60)
	Direct ^89^Zr labeling	CD19 CAR-T cells	K562 tumors in NSG mice	(61)
	PSMA + [^18^F]DCFPyL	CD19 CAR-T cells	ALL in mice	(62)
	TfR CAR-ΔPSMA reporter + [^68^Ga]Ga-PSMA-617	TfR CAR-ΔPSMA T cells	Breast cancer xenograft in mice	(63)
PET/NIRF	PET/NIRF nanotag	hCEA-redirected human CAR-T cells	Ovarian peritoneal carcinomatosis in mice	(65)
MPI/FMI	SPION/DiR dual labeling	CLDN18.2 CAR-T cells	HGC27 xenografts in NOD/SCID mice	(66)
FL	Single-cell optical imaging	EGFRvIII or EGFR CAR-T cells	Glioma and RMS xenografts in zebrafish	(67)
FL/PET	FL imaging + ^68^Ga-PSMA-617 PET	In situ-engineered CD19 CAR-T cells	B-cell lymphoma-bearing mice	(68)
GV-US	Gas vesicle-labeled CAR-T cells	CD19 CAR-T cells	Lymphoma in mice	(69)

### 4.2 Imaging for tumor recognition of CAR-T cells

**Figure 4:**
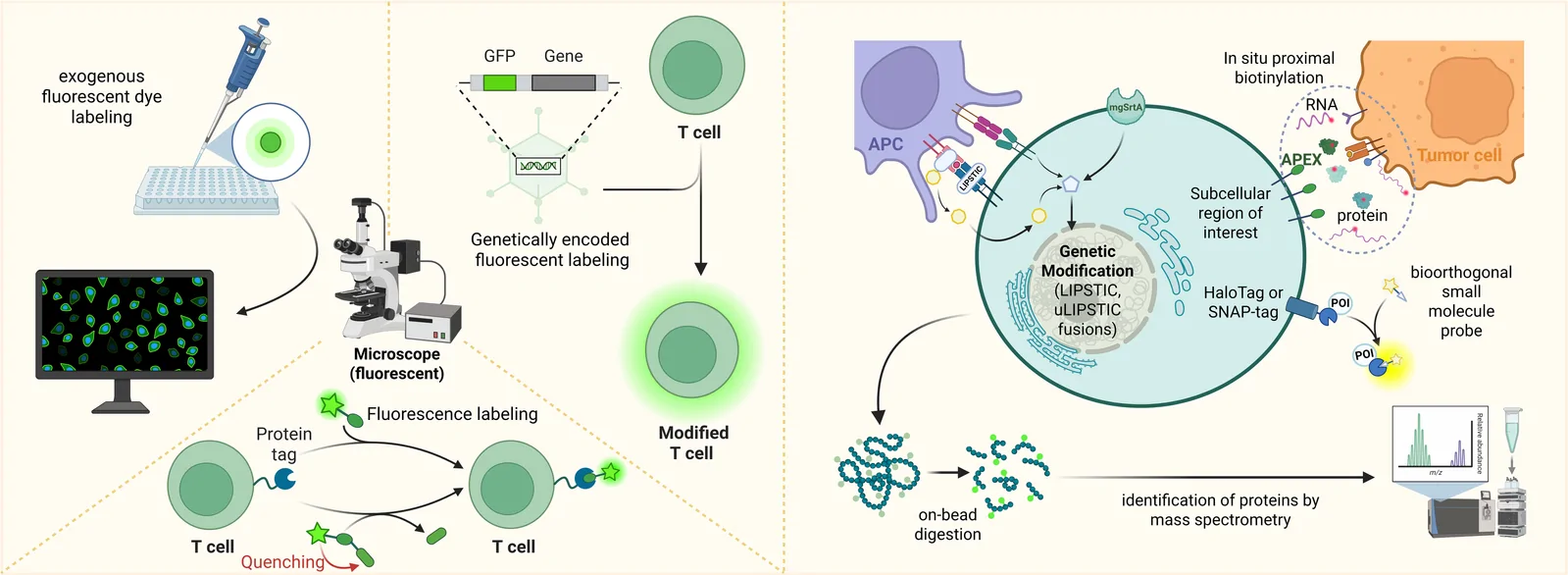
Imaging for tumor recognition of CAR-T cells. Advances in genetic engineering, fluorescence labeling, proximity labeling, and high-resolution imaging technologies have significantly enhanced the spatiotemporal characterization of T cell-tumor cell interactions. GFP, green fluorescent protein; POI, protein of interest. Figure created using BioRender (biorender.com).

Antigen-specific recognition of tumor cells by CAR-T cells constitutes a pivotal step in the orchestration of effective anti-tumor immune responses (70). Accordingly, real-time visualization of this recognition process via molecular imaging holds significant scientific importance. In recent years, molecular imaging has emerged as an indispensable tool for the noninvasive and dynamic monitoring of T cell-tumor cell interactions. Substantial advances in genetic engineering, fluorescence labeling, proximity labeling, and high-resolution optical microscopy have markedly expanded the capacity to characterize T cell-tumor cell interactions with enhanced spatiotemporal precision (71, 72) (**Figure 4**). These emerging imaging platforms provide critical mechanistic insights into anti-tumor immunity and offer valuable tools for evaluating immunotherapeutic efficacy.

#### 4.2.1 Fluorescence labeling and optical imaging

Fluorescence-based optical imaging serves as a prevalent technique for visualizing CAR-T cell-tumor cell interactions *in vivo*. Through fluorescent protein or dye labeling of CAR-T cells and tumor cells, this approach enables real-time observation of such interactions with high spatiotemporal resolution. When combined with methods including confocal microscopy and intravital imaging, it allows for the monitoring of T cell migration, tumor recognition, and cytotoxic activity, thereby providing insights into T cell-mediated anti-tumor responses. Among optical imaging platforms, multiphoton intravital microscopy is particularly valuable for resolving the dynamic behavior of immune cells within living tissues at cellular or subcellular resolution. Using two-photon imaging and a genetically encoded apoptosis reporter, Cazaux et al. observed rapid CAR-T-cell engagement and killing of lymphoma cells in the bone marrow, while demonstrating that not all cellular contacts elicited signaling or target-cell death, thereby revealing substantial functional and anatomical heterogeneity (73). In an orthotopic glioblastoma model, fluorescence-based intravital microscopy further visualized GD2 CAR-T-cell interactions with tumor cells and the resulting apoptosis, and showed that radiotherapy promoted productive CAR-T-cell accumulation within the tumor microenvironment (74). More recently, the Immunemap platform integrated 400 murine intravital microscopy videos and over 58,000 curated single-cell tracks across multiple tissues, providing a standardized resource for quantitative analysis of immune-cell motility *in vivo* (75). Collectively, these studies highlight the value of intravital optical imaging for direct, time-resolved assessment of CAR-T cell–tumor cell engagement and the associated cytotoxic response *in vivo*, although its application remains predominantly preclinical.

At the labeling level, genetically encoded fluorescent proteins have emerged as powerful tools for visualizing T cell–tumor cell interactions *in vivo* and dynamically tracking T-cell behavior during target-cell engagement. For example, Wang X. et al. fused the red fluorescent protein mCherry to the C-terminus of the CAR construct via a self-cleaving T2A peptide, while tumor cells were engineered to express GFP for discrimination (76). This dual-color system enabled real-time tracking of CAR-T cells and tumor cells, allowing visualization of their spatial relationships and assessment of CAR-T cell anti-tumor activity over time using time-lapse fluorescence microscopy. Additionally, genetically encoded fluorescent labeling enables dynamic visualization of protein expression and subcellular localization during immune cell recognition of tumor cells. By genetically fusing a fluorescent reporter or labeling tag to a protein of interest, this strategy allows real-time monitoring of changes in protein expression, intracellular trafficking, and protein-protein interactions. Although direct applications in CAR-T cells remain limited, representative studies in conventional cytotoxic T lymphocytes (CTLs) illustrate the potential of this approach. For example, Bednar et al. developed a fluorogenic granzyme B (GZMB)-responsive reporter that remains nonfluorescent until cleaved by GZMB released from CTLs upon antigen recognition, producing a strong and sustained fluorescence signal during target-cell killing (77). This strategy enables sensitive visualization of antigen-specific cytotoxicity and provides a valuable framework for monitoring the functional engagement of CAR-T cells during tumor recognition and elimination.

Similarly, exogenous fluorescent dye labeling has emerged as a flexible and convenient strategy for short-term visualization of immune cell interactions (78). He et al. established a transparent zebrafish metastasis model to directly visualize the *in vivo* cytotoxicity of human tumor-infiltrating lymphocytes (TILs) and CAR-T cells (79). By fluorescently labeling tumor cells with calcein AM and effector T cells with 1,1′-dioctadecyl-3,3,3′,3′-tetramethylindocarbocyanine perchlorate (DiI), they dynamically tracked intercellular interactions and quantified oncolysis at single-cell resolution. This model demonstrated potent, antigen-specific killing of both primary tumors and early microscopic metastases. Additional mechanistic insights have been obtained from studies in conventional CTLs. Using multicolor time-lapse confocal microscopy, Qi et al. visualized the sequential interactions between CTLs and B16 melanoma cells. Prior to imaging, CTLs were labeled with the fluorescent dye 5-(and-6)-carboxyfluorescein diacetate succinimidyl ester (CFSE), enabling real-time tracking of CTL behavior during tumor cell engagement. The results indicated that efficient tumor cell elimination is frequently associated with the formation of CTL clusters, which significantly enhances cytotoxic efficiency compared with individual CTL-tumor cell interactions (80). Although these findings were obtained in conventional CTLs rather than CAR-T cells, they provide important mechanistic insights into immune cell cooperation that may also inform the investigation of CAR-T-cell-mediated tumor killing.

#### 4.2.2 Proximity-labeling strategies

Although fluorescence imaging is well-suited for real-time visualization of dynamic cellular behaviors, it exhibits inherent limitations in preserving information regarding transient, weak, or low-affinity intercellular interactions. To address this constraint, proximity-labeling technologies have emerged as robust approaches for converting short-lived cell-cell contacts into stable biochemical tags. By harnessing enzymes or catalytic reactions that enable covalent labeling of adjacent biomolecules within confined spatial ranges, these strategies facilitate retrospective documentation of immune synapse formation and intercellular communication processes (81).

Representative applications of proximity-labeling technologies have largely been demonstrated in conventional immune-cell systems. For example, the MultiMap platform leverages a single photocatalyst to realize tunable, multi-scale labeling, enabling the characterization of both intracellular and intercellular interactomes, including immune synapses, without the requirement for genetic modification. This strategy thus provides a flexible framework for mapping spatially constrained molecular interactions in complex cellular environments. Similarly, LIPSTIC is an engineered enzyme-based proximity-labeling system that facilitates the recording of cell-cell receptor-ligand interactions in both *in vitro* and *in vivo* settings (82). By deploying a mutated Sortase A fused to interacting partners, this system catalyzes the covalent transfer of a detectable peptide substrate across immune synapses, thereby encoding interaction history onto receptor-expressing cells. This approach enables highly specific, genetically tractable, and *in vivo*-compatible detection of dynamic cellular interactions between CD4^+^ helper T cells and antigen-presenting cells, thereby establishing a proof-of-concept framework that could be adapted for dissecting CAR-T-cell interactions in future studies. Moreover, a universal variant version of LIPSTIC, designated as uLIPSTIC, has been further developed to record physical cell-cell interactions in both immune and non-immune contexts, independent of specific receptor-ligand pairs (83). uLIPSTIC has been applied across diverse biological scenarios, including the monitoring of dendritic cell-mediated CD8^+^ T cell priming, the identification of steady-state interaction partners of regulatory T cells, and the definition of cognate interactions between T follicular helper cells and germinal center B cells. When integrated with single-cell transcriptomics, uLIPSTIC further enables systematic mapping of immune-epithelial interactions in the intestine and dynamic profiling of virus-specific CD8^+^ T cell interactomes across multiple tissues during systemic infection. These features render uLIPSTIC a broadly applicable platform for interrogating cell-cell communication across complex biological systems. Beyond recording cell–cell encounters, proximity-labeling approaches can also be used to interrogate the molecular composition of contact interfaces. BioID, for example, enables biotin-dependent labeling of proteins surrounding a selected bait protein, followed by affinity enrichment and mass spectrometric identification, thereby providing a complementary proteomic readout rather than direct visualization of intercellular contacts (84). For direct optical interrogation of specific proteins at these interfaces, self-labeling tags such as SNAP-tag and HaloTag permit covalent fluorophore attachment, enabling dynamic imaging of key molecules involved in T cell–tumor cell interactions. Although primarily developed in conventional T-cell systems, these approaches provide a framework for visualizing molecular events during CAR-T-cell recognition and immune synapse formation. Their compatibility with super-resolution microscopy enables high-resolution imaging of cellular crosstalk (85).

Beyond genetically encoded enzyme-based systems, photocatalytic and bioorthogonal proximity-labeling approaches have recently emerged as versatile strategies for capturing transient T cell-tumor cell interactions in more physiologically relevant biological environments. By integrating photocatalysis with bioorthogonal chemistry, these methods enable rapid, proximity-dependent covalent labeling of interacting cells without requiring stable genetic modification, a feature that confers particular advantages for primary immune cells and clinically derived specimens. In the context of dynamic T cell-tumor cell interactions, Peng et al. developed a series of innovative proximity-labeling platforms, including EXCELL, CAT-Cell, and PhoXCELL (86–88). These systems are generally based on the non-genetic anchoring of photocatalysts (e.g., dibromofluorescein or iridium complexes) onto “bait” cells. Upon visible-light irradiation, these photocatalysts generate highly reactive short-lived intermediates that enable distance-dependent labeling of adjacent “prey” cells, thereby converting transient cellular contacts into stable chemical signatures. Among these approaches, PhoXCELL employs green-light-triggered singlet oxygen generation with a highly restricted diffusion radius (~30 nm), enabling high-spatiotemporal-resolution mapping of immunological synapses via oxidation-mediated protein labeling (87). To further expand labeling versatility, the authors introduced a quinone methide (QM)-based photocatalytic decaging strategy, in which blue-light activation generates tunable electrophilic intermediates capable of covalently modifying nucleophilic residues on neighboring cell surfaces. Importantly, modulation of QM diffusion kinetics allows quantitative assessment of interaction strength, including weak TCR-pMHC engagements that are otherwise difficult to capture. These studies establish proof-of-concept methodologies for resolving dynamic immune-cell interactions and may facilitate future investigation of CAR-T-cell recognition of tumor cells.

Collectively, photocatalytic bioorthogonal labeling systems address several critical limitations inherent to conventional genetic engineering approaches, particularly in the context of primary immune cells and clinical specimens. By transducing transient immune contacts into stable, sequenceable biochemical readouts compatible with single-cell omics platforms, these methodologies significantly expand the capacity to characterize functional immune interactions. However, their reliance on visible-light activation introduces intrinsic constraints on tissue penetration, thereby restricting applications in deep solid tumors and internal organs. Future advancements may thus prioritize the development of near-infrared-responsive photocatalysts, upconversion nanomaterials, and minimally invasive optical delivery systems to enable deep-tissue *in vivo* mapping of immune cell interactions (86). While these emerging technologies have been extended to three-dimensional organoid systems and superficial tissue imaging, most remain restricted to preclinical settings, with limited clinical translation to date (89, 90). Therefore, the realization of real-time, dynamic, and high-resolution visualization of T cell-tumor interactions *in vivo* remains a major challenge and a critical frontier in molecular imaging. Extending these technologies to CAR-T-cell-specific applications will be an important direction for future research (**Table 3**).

**Table 3:** Imaging for CAR-T-cell/T-cell–tumor cell recognition.

Strategy	Representative technique	Molecular component	Interaction interrogated	Refs
Fluorescence/optical imaging	Two-photon intravital microscopy with an apoptosis reporter	Fluorescent CAR-T cells; CFP–DEVD–YFP caspase-3 FRET reporter	CAR-T–tumor cell engagement, target-cell apoptosis, and contact-outcome heterogeneity *in vivo*	(73)
	Longitudinal fluorescence-based intravital microscopy through a cranial window	Fluorescent GD2 CAR-T cells; GFP-expressing glioma cells	CAR-T–tumor cell engagement and tumor-cell apoptosis in orthotopic glioblastoma	(74)
	Dual-color fluorescent-protein labeling	mCherry; GFP	CAR-T-tumor cell contact, spatial relationship, and anti-tumor activity	(76)
	GZMB-responsive fluorescent reporter	GZMB-cleavable dark-to-bright biosensor	Antigen-specific cytotoxic engagement and tumor-cell killing	(77)
	Transparent zebrafish fluorescence platform	Calcein AM; DiI	TIL/CAR-T-tumor cell engagement and single-cell oncolysis	(79)
	Multicolor time-lapse confocal microscopy	CFSE	CTL-tumor cell sequential interaction and cluster-associated killing	(80)
Proximity-labeling strategies	MultiMap	Single photocatalyst; proximity probes	Immune synapse-associated intercellular proximity interactions	(71)
	LIPSTIC	Sortase A; peptide substrate	Defined receptor-ligand-dependent cell-cell contacts	(82)
	uLIPSTIC	Membrane-anchored Sortase A	General physical T cell-target cell contacts	(83)
	SNAP/HaloTag-based fluorogenic labeling	SNAP-tag; HaloTag	Dynamic imaging of immune synapse-related proteins and cellular crosstalk	(85)
	EXCELL	Engineered sortase A (mgSrtA) and peptide substrate	Direct cell–cell interactions under living conditions	(88)
	PhoXCELL	Singlet oxygen system	Immune synapse formation at high spatiotemporal resolution	(87)
	CAT-Cell (QM-based photocatalytic decaging)	Photocatalyst and caged QM precursor	TCR–pMHC and tumor–T cell interactions	(86)

### 4.3 Imaging for CAR-T cell activity

The therapeutic efficacy of CAR-T cells ultimately depends on their ability to retain functional activity following tumor infiltration and target engagement. Because direct CAR-T-specific evidence for several activity-oriented imaging strategies remains limited, representative studies in conventional T cells and non-CAR immunotherapy are discussed as proof-of-concept evidence rather than direct clinical validation. As a key determinant of antitumor efficacy, T-cell functional activity also represents a valuable imaging biomarker for therapeutic assessment and response prediction. A comprehensive review of *in vivo* molecular imaging strategies targeting T cell activity has been previously presented in our work, which can be broadly categorized into metabolism-associated imaging, redox-associated imaging, surface receptor-associated imaging, and imaging of secreted molecules or functional outputs (91) (**Table 4**).

**Table 4:** Representative molecular imaging strategies for monitoring T cell activity *in vivo*.

Imaging strategy	Target/biomarker	Representative probe/modality	Key feature	Refs
Metabolism-associated imaging	Glycolytic flux; dCK/dGK axis	Hyperpolarized [1-^13^C]pyruvate MRI; [^18^F]F-AraG PET	Reflects metabolic reprogramming	(92–95)
Redox-associated imaging	ROS; redox homeostasis	T-Fulips MRI; SPNRs	Indicates redox stress and functional integrity	(96, 97)
Surface receptor-associated imaging	OX40, ICOS, CD69; PD-1, LAG-3	OX40 PET; ICOS/CD69 immunoPET; PD-1/LAG-3 PET or SPECT	Reflects activation, inhibition, or exhaustion	(98–103)
Secreted molecules/functional outputs	GZMB, IL-2, IFN-γ,CXCL9	GZMB PET; IL-2 imaging; CXCL9 PET	Effector-associated readout	(104–110)

dCK, deoxycytidine kinase; dGK, deoxyguanosine kinase; F-AraG, fluorinated arabinosyl guanine; ICOS, inducible T-cell co-stimulator; IFN-gamma, interferon-gamma; IL-2, interleukin-2; ROS, reactive oxygen species; SPNRs, semiconducting polymer nanoreporters. These categories summarize functional-state readouts and should be interpreted in relation to timing, cellular specificity, and the tumor microenvironment.

Metabolism-associated imaging targets glycolysis-related metabolites and the deoxycytidine kinase/deoxyguanosine kinase (dCK/dGK) axis, with representative modalities including hyperpolarized [1-^13^C]pyruvate magnetic resonance imaging (MRI), [^18^F]F-AraG and dCK-PET (92, 93). Recent studies indicate that this category enables the capture of immune activation at both systemic and regional levels, as evidenced by the correlation between vertebral bone marrow [^18^F]F-AraG uptake and survival outcomes in non-small cell lung cancer (NSCLC) patients receiving anti-PD-L1 therapy, and by the detection of tumor-draining lymph node activation via dCK-PET following immune checkpoint blockade (94, 95). Redox-associated imaging, exemplified by the T-Fulips MRI platform and semiconducting polymer nanoreporters (SPNRs), offers complementary insights into redox stress and functional integrity, but remains predominantly in the preclinical stage (96, 97). Surface receptor-associated imaging focuses on phenotypic markers linked to activation, inhibition, or exhaustion, including OX40, ICOS, CD69, PD-1, and LAG-3, through modalities such as OX40 PET, ICOS immunoPET, CD69 immunoPET, PD-1 PET, and LAG-3 PET/SPECT (98–103). In this area, LAG-3 has emerged as a promising target for immune monitoring, with LAG-3 imaging supporting early response assessment and dual-target imaging of LAG-3 and PD-L1 enabling noninvasive immunotyping of lung cancers (98, 99). At the level of functional output, molecular imaging of secreted molecules targets factors including GZMB, IL-2, IFN-γ, and CXCL9 (104–109). Recent advancements have been particularly prominent for GZMB, as evidence from both clinical PET imaging and activity-responsive NIR-II fluorescence probes validates its utility as a functional readout for the early evaluation of immunotherapy responses (107, 108). Furthermore, CXCL9-based PET imaging suggests that functional imaging can integrate microenvironment-derived signals associated with T cell activity (110). Collectively, these developments demonstrate that molecular imaging of T cell activity is transitioning from generalized functional inference to more specific and clinically relevant assessments of whether T cells are effectively exerting anti-tumor effects.

## 5 Discussion and perspective

Despite the remarkable clinical achievements of CAR-T cell therapy in hematological malignancies, its translation to solid tumors remains significantly constrained by a series of interconnected biological barriers, including inefficient tumor trafficking, limited intratumoral infiltration, antigen heterogeneity, an immunosuppressive TME, and progressive T cell exhaustion. As systematically outlined in this review, these challenges are intrinsically linked to the *in vivo* fate of CAR-T cells, specifically their spatial distribution, tumor recognition capacity, and real-time functional status. Therefore, elucidating the post-infusion behavior of CAR-T cells is not merely a mechanistic inquiry but a prerequisite for enhancing therapeutic efficacy and accelerating clinical translation. Molecular imaging has emerged as an indispensable platform to address this challenge by enabling noninvasive, dynamic, and quantitative monitoring of CAR-T cells *in vivo*. Conventional imaging paradigms have largely focused on tracking the biodistribution and tumor accumulation of CAR-T cells using modalities such as PET, MRI, fluorescence imaging, MPI, and ultrasound. While these approaches provide valuable insights into cell trafficking and persistence, they share a fundamental limitation: the presence of CAR-T cells at tumor sites does not necessarily correlate with effective antitumor activity. Infiltrating CAR-T cells may fail to recognize target cells due to antigen loss or heterogeneity, become functionally exhausted under chronic stimulation, or be suppressed by inhibitory signals within the TME. Consequently, reliance solely on distribution-based readouts risks substantial overestimation of therapeutic efficacy.

In response to these limitations, recent advances in molecular imaging are progressively driving a paradigm shift in the field, transitioning from passive cell tracking to functional interrogation of CAR-T cell behavior. Emerging strategies now enable the visualization of CAR-T cell-tumor cell interactions, immunological synapse formation, and cytotoxic effector functions, thereby facilitating a more direct assessment of the active engagement between infiltrating CAR-T cells and tumor cells. Concurrently, activity-oriented imaging approaches targeting metabolic reprogramming, activation-associated surface receptors, and effector molecules yield a multidimensional readout of CAR-T cell functional states within the TME. These developments highlight a critical paradigm shift: future imaging strategies must integrate information across multiple biological dimensions, including localization, recognition, and activity, to achieve a truly comprehensive evaluation of CAR-T therapeutic responses. Despite these advancements, several critical challenges persist in hindering the broader clinical application of molecular imaging in CAR-T cell therapy. Direct cell-labeling approaches, while operationally straightforward, are plagued by signal dilution during cell proliferation, potential probe leakage, and the risk of label transfer to non-target cells (e.g., phagocytes), which compromises imaging specificity and quantitative accuracy. Reporter gene strategies offer enhanced specificity and enable longitudinal monitoring, but they necessitate additional genetic modification of CAR-T cells, introducing regulatory and safety considerations for clinical translation. Furthermore, many recognition- and activity-based imaging modalities remain restricted to preclinical settings due to limitations such as poor tissue penetration (e.g., fluorescence-based approaches), insufficient sensitivity for rare cellular events, or the complexity associated with probe design and synthesis (**Table 5**).

**Table 5:** Comparative translational feasibility of various imaging strategies.

Imaging strategy	Representative approaches	Infrastructure	Regulatory/safety considerations	Cell types	Translational status	Refs
Tracking and biodistribution						
MRI-based cell tracking	Gadolinium-based probes (e.g., NaGdF4-TAT nanoparticles)	MRI scanner	Gadolinium/label toxicity; signal dilution; limited viability assessment	Adoptive T cells	Preclinical	(50)
	Iron oxide nanoparticles (SPIONs/USPIOs, ferumoxytol, MegaPro-NPs; optional MRI/PAT/MPI readout)	MRI scanner; optional PAT or MPI scanner	Nanoparticle toxicity or hypersensitivity; persistence after cell death; signal dilution	CAR-T cells	Preclinical	(51–53)
	^19^F-labeled perfluorocarbon nanoemulsions	Multinuclear MRI scanner with a dedicated ^19^F coil	Specialized hardware; limited sensitivity; label dilution with proliferation	CAR-T cells	Preclinical	(54)
Direct radionuclide labeling	^89^Zr-oxine or direct 89Zr labeling	PET/CT scanner	Radiation burden; label efflux; signal dilution; inability to confirm cell viability	CAR-T cells	Preclinical	(58, 61)
	[^68^Ga]Ga-DOTA-biotin or 68Ga-oxine/direct 68Ga labeling	PET/CT scanner	Radiation dosimetry; short imaging window; label efflux and dilution	CD19 CAR-T cells	Preclinical	(59, 60)
Nuclear reporter gene imaging	PSMA reporters with [^18^F]DCFPyL or [^68^Ga]Ga-PSMA-617	PET/CT scanner	Stable gene transfer; ectopic reporter expression; tracer approval; long-term follow-up	CAR-T cells	Preclinical	(62, 63)
Fluorescence-based cell tracking	PET/NIRF fluorescent nanotags	PET/CT and NIR fluorescence imaging systems	Dual-probe regulation; radiation exposure; probe stability and retention	CAR-T cells	Preclinical	(65)
	DiR/SPION dual labeling; fluorescent-protein or single-cell optical imaging; FL/PET hybrid imaging	Fluorescence microscope; PET/CT or MPI scanner	Dye leakage or transfer; signal dilution; phototoxicity; limited tissue penetration	CLDN18.2-, EGFR/EGFRvIII-, or CD19-targeted CAR-T cells	Preclinical	(66–68)
Ultrasound-based cell tracking	Gas-vesicle-labeled CAR-T cells	Ultrasound imaging system	Label biocompatibility and immunogenicity; signal stability; preservation of cell function	CD19 CAR-T cells	Preclinical	(69)
Magnetic particle imaging	SPION-labeled CAR-T cells, including MPI/FMI dual-modality tracking	MPI scanner; optional fluorescence imaging system	Particle persistence; uncertain correspondence with cell viability; limited clinical equipment	CLDN18.2 CAR-T cells	Preclinical	(66)
Tumor recognition and cell–cell interaction						
Intravital optical microscopy	Two-photon imaging with an apoptosis reporter; longitudinal cranial-window fluorescence imaging	Multiphoton intravital microscope	Limited depth and field of view; phototoxicity; invasive access in many organs; genetic labeling	CAR-T cells and tumor cells	Preclinical	(73, 74)
Fluorescence/optical interaction imaging	Dual-color fluorescent-protein labeling (mCherry/GFP)	Time-lapse confocal fluorescence microscope	Genetic modification; phototoxicity; limited tissue penetration	CAR-T cells and tumor cells	Preclinical	(76)
	GZMB-cleavable dark-to-bright fluorescent reporter	Fluorescence microscope	Probe specificity and toxicity; background activation; limited imaging depth	CTLs and target cells	Preclinical	(77)
	Transparent-zebrafish imaging with Calcein AM/DiI; CFSE-based time-lapse confocal microscopy	Confocal fluorescence microscope	Dye leakage or transfer; signal dilution; phototoxicity	CAR-T cells, TILs, CTLs, and tumor cells	Preclinical	(79, 80)
Proximity-labeling and interaction-recording strategies	MultiMap	Light irradiation system; flow cytometer; LC-MS/MS system	Reactive intermediates; off-target labeling; limited tissue penetration	CAR-T cells and broader interacting-cell models	Preclinical	(71)
	LIPSTIC and uLIPSTIC	Flow cytometer; single-cell sequencing platform	Genetic engineering; contact specificity; workflow complexity	CD4^+^/CD8^+^ T cells and antigen-presenting cells	Preclinical	(82, 83)
	SNAP/HaloTag fluorogenic labeling and EXCELL	Fluorescence or super-resolution microscope; flow cytometer	Genetic modification; nonspecific labeling; probe and substrate biocompatibility	General tagged cells and interacting cells	Preclinical	(85, 88)
	CAT-Cell (QM-based photocatalytic decaging) and PhoXCELL	Light irradiation system; flow cytometer	Phototoxicity; reactive species; off-target labeling; shallow tissue penetration	Antigen-specific T cells and dendritic or tumor cells	Preclinical	(86, 87)
Functional activity						
Metabolism-associated imaging	Hyperpolarized [1-^13^C]pyruvate MRI	MRI scanner; dynamic nuclear polarization system	Specialized equipment; short-lived signal; limited cellular specificity	Alloreactive CD4^+^ effector-memory T cells	Preclinical	(92)
	dCK-PET	PET/CT scanner	Tracer approval and dosimetry; metabolic background; limited CAR-T specificity	Activated T cells	Preclinical	(94)
	[^18^F]F-AraG PET	PET/CT scanner	Tracer approval and dosimetry; activated-T-cell rather than CAR-T specificity	Activated T cells	Clinical investigation (NCT04726215)	(95)
Redox-associated imaging	T-Fulips MRI	MRI scanner	Probe toxicity; redox specificity; signal reproducibility	Conventional T cells	Preclinical	(97)
	Semiconducting polymer nanoreporters (SPNRs)	Near-infrared chemiluminescence imaging system	Nanomaterial safety; phototoxicity; limited tissue penetration	Activated immune cells	Preclinical	(96)
Surface receptor-associated imaging	OX40 PET; ICOS and CD69 immunoPET	PET/CT scanner	Tracer approval; target perturbation; off-target expression; limited CAR-T specificity	Activated conventional T cells/TILs	Preclinical	(101–103)
	PD-1 PET and LAG-3 PET/SPECT	PET/CT scanner; SPECT/CT scanner	Checkpoint binding may alter biology; background uptake; limited CAR-T specificity	PD-1/LAG-3-expressing TILs	Clinical investigation (NCT02760225/NCT03780725)	(98–100)
Secreted effector and functional-output imaging	GZMB PET	PET/CT scanner	Tracer approval and dosimetry; extracellular background; limited CAR-T specificity	Cytotoxic T cells	Clinical investigation (NCT05000372)	(108, 109)
	Radiolabeled IL-2 imaging	SPECT/CT scanner; PET/CT scanner	Potential bioactivity of labeled cytokine; receptor specificity; dosimetry	Activated T cells/TILs	Clinical investigation (NCT02922283)	(104, 105)
	IFN-gamma imaging and CXCL9 PET	PET/CT scanner; SPECT/CT scanner	Low target abundance; background signal; tracer specificity and validation	Activated T cells	Preclinical	(106, 110)

The “Cell types” column summarizes the cell populations directly examined in the cited studies and distinguishes evidence derived from CAR-T cells from that obtained in conventional T cells or other immune-cell populations. “Preclinical” denotes evidence limited to *in vitro* and/or animal studies. “Clinical investigation” denotes evaluation in human imaging studies, with ClinicalTrials.gov identifiers provided where available. None of the listed approaches is currently established for routine clinical monitoring of CAR-T-cell fate.

CAR-T, chimeric antigen receptor T cell; dCK, deoxycytidine kinase; FL, fluorescence; FMI, fluorescence molecular imaging; GMP, good manufacturing practice; GZMB, granzyme B; MPI, magnetic particle imaging; MRI, magnetic resonance imaging; NIRF, near-infrared fluorescence; PAT, photoacoustic tomography; PET, positron emission tomography; PFC, perfluorocarbon; PSMA, prostate-specific membrane antigen; SPION, superparamagnetic iron oxide nanoparticle; SPECT, single-photon emission computed tomography; USPIO, ultrasmall superparamagnetic iron oxide nanoparticle.

Looking forward, the next generation of molecular imaging for CAR-T cell therapy will likely be shaped by the integration of multimodal and multiscale imaging technologies. The combination of whole-body, high-sensitivity modalities such as PET with high-resolution optical imaging or activatable smart probes could enable the simultaneous visualization of CAR-T cell trafficking, tumor engagement, and effector function in the same subject. Furthermore, the incorporation of artificial intelligence (AI)-driven image analysis and radiomics may facilitate the extraction of predictive biomarkers from imaging data, enabling the early stratification of responders and non-responders. From a translational perspective, future efforts should prioritize the development of clinically compatible imaging probes with favorable safety profiles, standardized protocols for image acquisition and analysis, and validation in physiologically relevant patient-derived tumor models (e.g., organoids or patient-derived xenografts). Ultimately, a systems-level understanding of CAR-T cell fate enabled by next-generation molecular imaging will not only improve real-time therapeutic decision-making but also guide the rational design of next-generation CAR-T cells with enhanced tumor-homing, recognition, and resistance to exhaustion.
